# Single-Walled Carbon Nanotube-Reinforced PEDOT: PSS Hybrid Electrodes for High-Performance Ionic Electroactive Polymer Actuator

**DOI:** 10.3390/ma17102469

**Published:** 2024-05-20

**Authors:** Haoxiang Tao, Guangyao Hu, Shun Lu, Bing Li, Yongxing Zhang, Jie Ru

**Affiliations:** 1 Mechanical and Electrical Engineering, College of Energy and Power Engineering, Lanzhou University of Technology, Lanzhou 730050, China; 2 Key Laboratory of Green and Precise Synthetic Chemistry and Applications, Ministry of Education, School of Chemistry and Materials Science, Huaibei Normal University, Huaibei 235000, China; 3 Chongqing Institute of Green and Intelligent Technology, Chinese Academy of Sciences, Chongqing 400714, China; 4 Anhui Province Key Laboratory of Pollutant Sensitive Materials and Environmental Remediation, School of Physics and Electronic Information, Huaibei Normal University, Huaibei 235000, China

**Keywords:** ionic electroactive polymer, actuators, spray printing technique, electromechanical properties

## Abstract

Ionic electroactive polymer (iEAP) actuators are recognized as exceptional candidates for artificial muscle development, with significant potential applications in bionic robotics, space exploration, and biomedical fields. Here, we developed a new iEAP actuator utilizing high-purity single-walled carbon nanotubes (SWCNTs)-reinforced poly(3, 4-ethylenedioxythiophene)/poly(4-styrenesulfonate) (PEDOT: PSS, PP) hybrid electrodes and a Nafion/EMIBF_4_ ion-exchange membrane via a straightforward and efficient spray printing technique. The SWCNT/PP actuator exhibits significantly enhanced electric conductivity (262.9 S/cm) and specific capacitance (22.5 mF/cm^2^), benefitting from the synergistic effect between SWCNTs and PP. These improvements far surpass those observed in activated carbon aerogel bucky-gel-electrode-based actuators. Furthermore, we evaluated the electroactive behaviors of the SWCNT/PP actuator under alternating square-wave voltages (1–3 V) and frequencies (0.01–100 Hz). The results reveal a substantial bending displacement of 6.44 mm and a high bending strain of 0.61% (at 3 V, 0.1 Hz), along with a long operating stability of up to 10,000 cycles (at 2 V, 1 Hz). This study introduces a straightforward and efficient spray printing technique for the successful preparation of iEAP actuators with superior electrochemical and electromechanical properties as intended, which hold promise as artificial muscles in the field of bionic robotics.

## 1. Introduction

Electroactive polymer (EAP) actuators are garnering increasing research interest and attention across various fields of modern science and technology, primarily because of their notable ability to undergo substantial deformation in response to external electrical stimulus [1,2]. Consequently, EAP actuators are being widely acknowledged as exceptional candidates for the development of ‘artificial muscles’, holding significant potential for applications in bionic robotics, space exploration, and biomedical fields [3,4].

Ionic polymer metal composites (IPMCs), a typical type of ionic electroactive polymer (iEAP) material, are usually comprised of an inner ion-exchange polymer layer (like Nafion) and metal electrodes (like Pd and Pt) on both sides, generally achieved through electroless plating [4,5]. These actuators exhibit substantial deformation toward the anode side at relatively low voltages due to the swelling of the cathode side, driven by the flow of hydrated ions in the inner polymer layer toward the cathode side through ion nanochannels. However, the metal electrodes of IPMCs, produced through multiple electroless plating processes, typically possess a rough surface and poor adhesion to the Nafion layer, negatively impacting the deformation of IPMCs. Moreover, the electrodes are susceptible to damage and cracking after thousands of cyclic deformations, leading to issues such as easy peeling, reduced repeatability, and prompt relaxation. The deformation of IPMCs, including back relaxation in hydrated conditions and deformation attenuation in air, is too unstable to be controlled [6]. This instability significantly hampers their potential applications.

In engineering fields, iEAP actuators are urgently desired to operate durably and stably in the air. In 2005, Asaka’s group [7] reported a groundbreaking category of iEAP actuators designed to work durably and stably in the air. These innovative actuators are assembled through the hot-pressing of a polymeric membrane with ionic liquid positioned in two pieces of bucky-gel electrodes [8,9,10]. The electrodes are typically made via casting a blended dispersion comprising supporting polymers, nanoconductive materials, and ionic liquid. However, a large number of nonconductive polymers in the electrodes always results in poor conductivity compared with those of pure nanocarbon materials [10,11]. Additionally, their volumetric capacitance is somewhat inferior to that of supercapacitor electrodes [12,13]. These features lead to a delay in the electrochemical kinetic processes within the resulting actuators, ultimately requiring compromises in their actuation performances.

To further enhance actuation behaviors, numerous exceptional nanocarbon materials, such as hierarchal carbon nanotubes and graphene mesh, have been developed for iEAP actuators [14,15]. Due to the unique structure and electrochemical characteristics of the electrodes, the corresponding actuators exhibited significant increasements, with orders of magnitude greater power density compared with conventional bucky-gel actuators. Despite the enhanced actuation behaviors, the exploration of these exceptional nanomaterials is just on the laboratory scale. Their rare production and high cost significantly constrain the engineering applications of the resulting actuators.

Poly(3,4-ethylenedioxythiophene)/poly(4-styrenesulfonate) (PEDOT: PSS, PP), recognized as a promising conductive polymer, has been embraced as an alternative electrode for constructing iEAP actuators, leveraging its softness, high chemical stability, and electrical conductivity [16]. Typically, the following methods have been reported for preparing actuators with PP electrodes: spin-coating [17], dip-casting or drop-casting [10], and hot-pressing [4]. To our knowledge, electrode thickness is difficult to control in the spin-coating process at high speeds, with significant wastage of coating material. The dip or drop-casting methods have differences in evaporation rates on the substrate or concentration fluctuations, readily inducing uneven electrode surfaces. In the hot-pressing process, self-standing PP layers are initially required, yet the accessible PP in the market usually possesses poor film-forming capabilities. It should be noted that the conductivity and capacitance of the electrodes made from commercially available PP is somewhat low. This makes it often difficult to meet the requirements of iEAP actuators regarding the high electrical and electrochemical properties of electrode layers, thus forcing the actuation properties to be compromised.

In previous reports, it has been found that the electric and electrochemical properties of single-walled carbon nanotube/PP (SWCNT/PP) hybrid films can be significantly enhanced due to the synergistic effect between PP and SWCNTs. The entangled SWCNT/PP network with mesopores [18,19,20] facilitates easy access for ions. Simultaneously, the SWCNTs skeleton would enhance the conductivity and provide electrostatic double-layer capacitors (EDLC). This may result in a high degree of actuation behaviors for the corresponding actuators. However, the preparation methods employed in the above-mentioned studies are still not straightforward enough. The development of a simplified preparation method remains an urgent issue to be addressed.

Here, we employed a straightforward and efficient spray printing technique to create iEAP actuators with SWCNT/PP hybrid electrodes, capitalizing on the synergistic effect between PP and SWCNTs. The electrochemical and electromechanical performances of the SWCNT/PP actuator were systematically tested and compared with those of the PP actuator and some other previously reported iEAP actuators. Moreover, it is expected that the SWCNT/PP actuator would exhibit greater actuation behaviors than the PP actuator and other iEAP actuators. Therefore, we hope that the actuator developed here will find extensive applications in bionic robotic fields.

## 2. Experimental Section

### 2.1. Experimental Materials

Nafion solution (DE-520, 20 wt%) was purchased from DuPont Company (Shanghai, China). The high-purity SWCNTs were purchased from Chengdu Organic Chemical Co. Ltd. (Chengdu, China). Table 1 shows the physical properties of the SWCNTs. PP (1.1 wt% in water), 1-Ethyl-3-methylimidazolium tetrafluoroborate (EMImBF_4_, 99.9 wt%), N,N-dimethylacetamide (DMAc, 99 wt%), and anhydrous ethanol (EtOH, 99.5 wt%) were purchased from Aladdin (Shanghai, China). All the reagents were used as received.

### 2.2. Preparation of Nafion/EMImBF_4_ Membrane

An amount of 1.25 g of Nafion solution (20 wt%), 0.25 g EMImBF_4_, 9.25 g DMAc, and 3.75 g H_2_O were put into a glass sample bottle and continuously stirred for 4 h to prepare Nafion/EMImBF_4_ casting solution. A total of 11.12 g of the casting solution was poured into a glass container (50 mm × 50 mm × 30 mm) and evaporated thoroughly at 70 °C in open air to prepare the Nafion/EMImBF_4_ membrane. Then, the membrane was put into an oven to dry at 80 °C and 120 °C for 2 h, respectively, and finally treated at 150 °C for 30 min. The size of the prepared Nafion/EMImBF_4_ membrane was 50 mm × 50 mm × (70 ± 5) μm (length × width × thickness).

### 2.3. Fabrication of Actuators

(a) Preparation of spraying solutions: An amount of 0.08 g of SWCNTs was dispersed into 16 mL of EtOH by sonicating in an ice-water bath for 45 min to prepare SWCNT/EtOH dispersion. An amount of 0.55 g of PP solution (1.1 wt% in water) was dissolved into 12 mL of EtOH and stirred for 2 h to obtain the PP/EtOH solution. Then, 6 mL of SWCNT/EtOH dispersion and 6 mL of PP/EtOH solution were mixed and stirred for 2 h to prepare the SWCNT/PP/EtOH solution.
(b) Preparation of actuators: First, a 2 cm × 1.5 cm sized Nafion/EMImBF_4_ membrane was fixed on a heating platform. Second, 0.1 mL of the casting solution was sprayed onto the membrane surface using a spray gun. The platform was heated to 120 °C to completely remove DMAc and other solvents. After that, 12 mL of the SWCNTs/PP/EtOH solution was put into the spray gun and sprayed onto the membrane surface. The membrane was then heated to 80 °C to remove EtOH and to form a conductive surface, which works as an electrode while the actuator is working. After that, the above steps were repeated to prepare the electrode on the other side of the Nafion/EMImBF_4_ membrane. Finally, actuators with SWCNT/PP electrodes on both sides of the Nafion/EMImBF_4_ membrane were successfully obtained, which are named SWCNT/PP actuators. To be compared, actuators with PP electrodes were also prepared by utilizing the same method and named PP actuators. The actuators were cut into 20 mm × 2 mm × (95 ± 5) μm (length × width × thickness) sized specimens for characterization.

### 2.4. Characterizations

Morphological observation: Micromorphologies of the actuators were observed employing a field emission scanning electron microscope (FE-SEM, S-8200, Hitachi, Tokyo, Japan) to observe the cross-sectional and interfacial structures of the actuators and the microstructures of the electrodes.

The actuators’ electrochemical properties were evaluated by measuring the Cyclic Voltammetry (CV) and Electrochemical Impedance Spectroscopy (EIS) with an electrochemical workstation (CHI 660E, Chenhua, China) via two-electrode configuration. The electrodes’ surface resistances were measured using a four-probe resistance tester.

**Figure 1:**
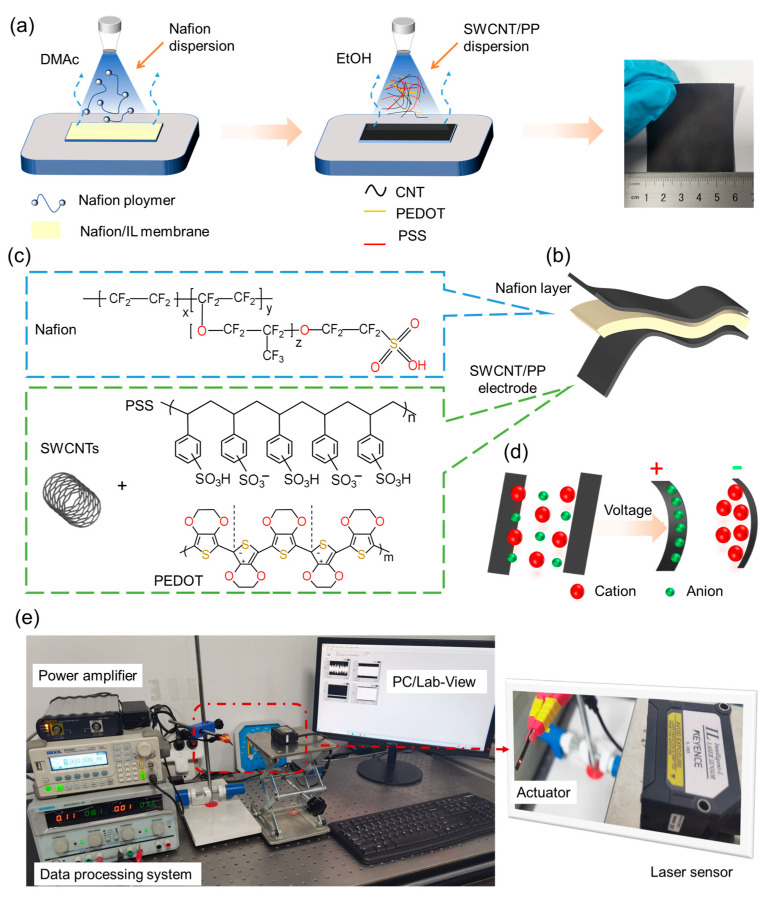
Configuration of the SWCNT/PP actuator: (**a**) Diagram for SWCNT/PP hybrid electrode preparation. (**b**) Image of a SWCNT/PP actuator. (**c**) Chemical formulas of Nafion, SWCNTs, and PP. (**d**) Illustration for actuation mechanism. (**e**) Testing platform for electromechanical actuation performances.

Electromechanical properties of the actuators were investigated by measuring the deformation using the test apparatus as shown in Figure 1e. The actuators were submitted to various voltages by a power supply (HM8143) and Labview (Labview 2020, National Instruments, USA). A laser displacement tester (LK-G80, Keyence, Osaka, Japan) was used to record the bending displacement at the 10 mm distance point. Three samples of each kind of actuator were tested parallelly. And the standard deviations of the recorded displacement were no more than 20%.

## 3. Results and Discussion

### 3.1. Morphological Observation

**Figure 2:**
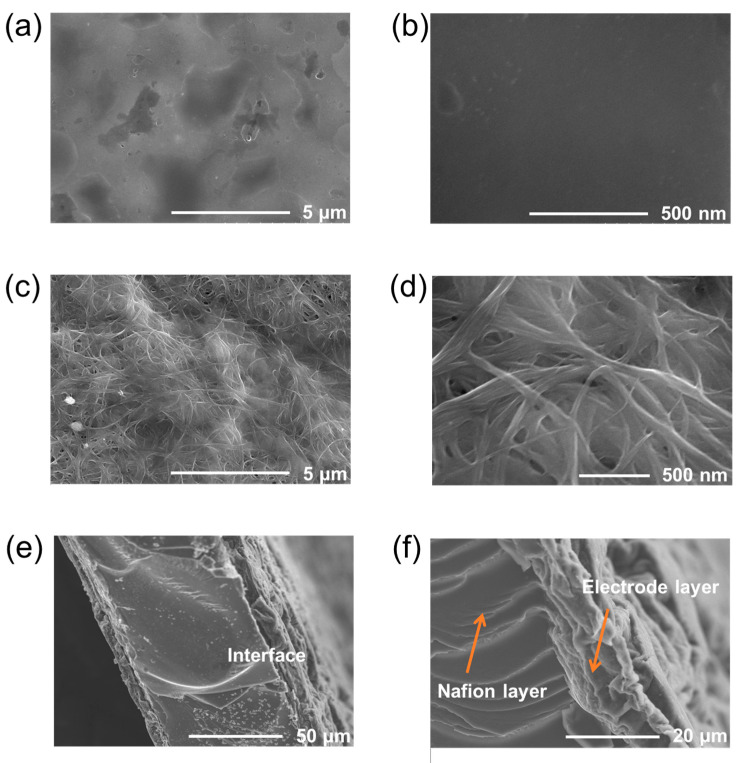
SEM images of SWCNT/PP electrode surface (**a**,**b**), SWCNT/PP electrode surface (**c**,**d**), and SWCNT/PP actuator’s cross-section (**e**,**f**) at different magnification levels.

The electrode surface microstructures and the actuator cross-sections were observed using a FE-SEM. In Figure 2a,b, the pure PP electrode exhibited a typical polymer surface. Upon incorporation of SWCNTs with PP, the SWCNT/PP electrode displayed a relatively rough surface with mesopore structures, where SWCNTs were well entangled by PP chains and homogeneously dispersed within PP polymers (Figure 2c,d). The rough microfibrous surfaces of the SWCNT/PP electrode increase its contact area with the inner Nafion membrane, while the porous structure vastly accelerates charge injection and ion migration among the actuators. These factors could enable the actuators to generate large deformation. As shown in Figure 2e, the SWCNT/PP actuator showed a typical sandwiched structure. More clearly depicted in Figure 2f, the SWCNT/PP electrode exhibited an average thickness of 13 μm and adhered well to the Nafion membrane without delamination. This close-knit adherence accelerates both the inter- and intralayer ion migration among the actuators, thereby enhancing their electromechanical actuation properties.

### 3.2. Electrochemical Characterizations

In conductivity testing, the SWCNT/PP actuator exhibited a surface conductivity of approximately 262.9 S/cm, whereas the PP actuator demonstrated a surface conductivity of only about 23.93 S/cm. It has been verified that PP possesses an adjustable conductivity, ranging from 0.1 to 3000 S/cm [21], and the addition of certain nanoconductive materials (such as CNTs and graphene) can significantly enhance the conductivity of PP. This means that the surface conductivity of the SWCNT/PP actuator was notably enhanced by integrating high-purity SWCNTs with high conductivity into the PP electrode. The heightened conductivity of the SWCNT/PP electrode ensures a sufficient electric field across the actuator and minimizes potential drop along the sample strip. Consequently, the actuator can function akin to a parallel-plate capacitor, facilitating electrochemical kinetics-related procedures [22,23].

**Figure 3:**
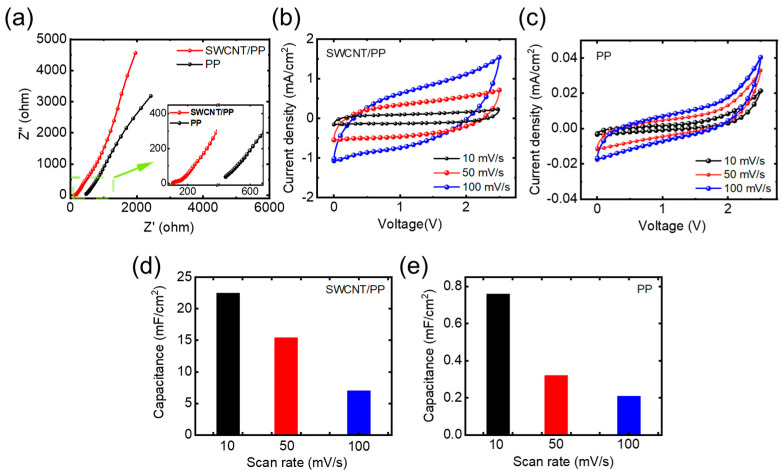
Electrochemical performances: (**a**) Nyquist plots, (**b**) and (**c**) CV profiles, (**d**,**e**) the calculated specific capacitance of the actuators.

The electrode impedance plays a pivotal role in determining the electromechanical performance of actuators. It is noteworthy that the characteristic impedance at low frequency is governed by the ion diffusion process, whereas the characteristic impedance at high frequency is dictated by the charge transfer process. The Nyquist plot in Figure 3a illustrates the real part of impedance (Z′ (f)) of the fabricated actuators, indicating that the SWCNT/PP actuator exhibits significantly lower impedance compared with that of the PP actuator. This reduction in impedance of the actuator primarily stems from the cooperative effect between SWCNTs and PP in the hybrid electrode.

From Figure 3b,c, we can see that there are no redox peaks in the CV curves. This revealed that the actuators possess a representative capacitive charge/discharge characteristic. The specific capacitance was calculated from the CV curves according to Equation (1):

(1) $$C=\frac{1}{(∆V)\times \vartheta }\int idV\;$$

where *C* is donated as areal capacitance, ∆*V* is the effective potential window, *i* is the current, and ϑ is the scan rate. The specific capacitances at various scan rates were calculated and presented in Figure 3d,e. These results indicate that the SWCNT/PP actuator represents significantly improved specific capacitances at various scan rates compared with those of the PP actuator. Notably, at 10 mV/s scan rate, the specific capacitance of the SWCNT/PP actuator is definitely higher than those of the actuators based on nanocarbon material bucky-gel electrodes [4,7].

The specific capacitances of the SWCNT/PP actuator are believed to arise from both the electrostatic double-layer capacitor (EDLC) mechanism of SWCNTs and the faradaic capacitor (FC) mechanism of PP, with the former contributing the most significantly. This contrasts with a conventional PP electrode, where FC is the sole mechanism. Additionally, the SWCNTs’ skeleton can not only enhance electrical conductivity but also afford the actuator an EDLC. Furthermore, the highly entangled SWCNTs and PP take the shape of a network of highly interconnected open mesopores [10,17,18], indicating that the SWCNT/PP electrode can be more easily accessed by ions. These phenomena illustrate the synergistic effect between SWCNTs and PP. Such enhancements in electrochemical properties would consequently bring forth superior electro-active behaviors for the SWCNT/PP actuators because ion migration is the key mechanism of electromechanical energy conversion.

### 3.3. Electromechanical Properties

**Figure 4:**
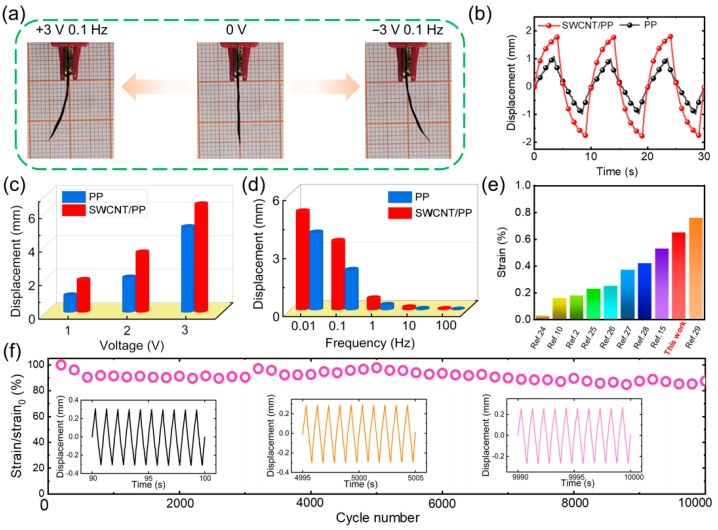
Electromechanical actuation characteristics: (**a**) Photograph of the actuator’s bending deformation under 3 V 0.1 Hz square-wave voltage. (**b**) Bending curves of the actuators under 2 V 0.1 Hz square-wave voltage. (**c**) Displacement under different applied amplitude. (**d**) Displacement under different applied frequencies. (**e**) A comparison of the iEAPactuators’ bending strain. (**f**) Actuation durability of the PP actuator under 2 V 1 Hz square-wave voltage.

When applied to an electric field, charge and discharge processes will occur separately inside the actuator. Subsequently, cations migrate to and accumulate at the cathode side, while anions migrate to and accumulate at the opposite side. Due to the geometric effect of EMIm^+^ and BF_4_^−^, the cathode expands much more largely than the anode does. Consequently, the actuator will bend toward the anode side (Figure 1d). To investigate the electromechanical behaviors, we recorded the displacement of the actuators under applied square-wave voltages (Figure 4a).

The deformation curves shown in Figure 4b clearly demonstrate that, as the actuator submits to a periodic voltage, the strain increases and decreases in accordance with the voltage variation, leading to a periodic deformation. All deformation curves keep the shapes in time, showing great stability and controllability for the actuators. Figure 4c illustrates that bending displacement significantly increases with higher applied voltage. According to the deformation mechanism of the capacitor-type actuator (Figure 1d), higher voltage results in greater charge accumulation on the electrodes, leading to increased migration of working ions in the Nafion membrane towards the electrodes, thus causing more swelling and larger bending displacement. Additionally, Figure 4d demonstrates that the bending displacement of the actuator decreases markedly when the frequency increases, as lower applied frequencies allow more time for bending to occur.

Furthermore, it is evident that the bending displacement and initial deformation slope under various applied voltages of the PP actuator significantly exceeded those of the actuator without SWCNTs, as shown in Figure 4c,d. For example, the SWCNT/PP actuator exhibited bending displacements of 1.93, 2.07, and 5.08 under 0.1 Hz square-wave voltages with amplitudes of 1 V, 2 V, and 3 V, respectively. In contrast, the PP actuator demonstrated bending displacements of 1.01, 2.07, and 5.08 under the same voltages. Such remarkable enhancements in the actuation performances of the SWCNT/PP actuator can greatly reveal the potentiation effects of SWCNTs doping in the PP electrode on the deformation behaviors. This is because the doping of SWCNTs with high conductivity and EDLC somewhat improves the conductivity and capacitance of the PP electrode. Consequently, the higher conductivity of the SWCNT/PP electrode enhances the charging efficiency and capacity of the corresponding actuators, further facilitating their bending deformation.

To compare the SWCNT/PP actuators’ actuation properties with those of the reported iEAP actuators, we calculated the strain (*ε*) using Equation (2):

(2) $$\varepsilon =\frac{2d\delta }{L^{2}+\delta^{2}}$$

where *δ*, *d*, and *L* represent the displacement, thickness, and measuring distance of the actuators, respectively. In the calculation, the SWCNT/PP actuator showed a strain of 0.61% under 3 V, 0.1 Hz square-wave voltage, marking a great increase of 28% compared with that of the PP actuator (0.47%). In Figure 4e, a comparison of the SWCNT/PP actuator’s strain with that of the reported iEAP actuators is presented. Remarkably, the strain of the SWCNT/PP actuator is comparable to or even higher than most of the reported iEAP actuators made from PP, SWCNTs, conductive polymers, graphene, and CNTs/gold electrodes [24,25,26,27,28,29]. This can be attributed primarily to the excellent properties of the SWCNT/PP electrode, such as excellent film-forming ability, high conductivity, and high capacitance.

The SWCNT/PP actuator has also demonstrated relative durability in air. Figure 4f presents the actuator’s cycling bending curves under ± 2 V, 1 Hz square-wave voltage in air. The actuator continuously operated up to 10,000 cycles, with only a 15% initial drop. This is mainly caused by the annealing of the actuator components. Nevertheless, the cycling operation of the SWCNT/PP actuator stays significantly stable in comparison with that of the actuators based on Pt or rGO electrodes [10,16].

## 4. Conclusions

In summary, to optimize the preparation process and electromechanical properties of iEAP actuators and thereby advance their applications in engineering fields, we developed an efficient and straightforward spray printing technique for fabricating iEAP actuators employing high-purity SWCNTs-reinforced PP hybrid electrodes and ion-exchange membrane. Benefiting from the synergistic effect between SWCNTs and PP, the electric conductivity (262.9 S/cm) and specific capacitance (22.5 mF/cm^2^) of the SWCNT/PP actuator were significantly improved compared with those of the PP actuator because of the high conductivity and enhanced EDLC provided by the incorporated SWCNTs. Due to the enhanced performance of the hybrid electrode, the SWCNT/PP actuator exhibited a substantial bending displacement of 6.44 mm and a large bending strain of 0.61% (at 3 V, 0.1 Hz) with a long life cycling stability of more than 10,000 cycles (at 2 V, 1 Hz), surpassing the performances of the PP actuator in this study and most previously reported iEAP actuators. Benefiting from these significant achievements, we hope that the newly developed actuators will serve as alternative candidates with great promise for artificial muscles. Moreover, the simple and efficient spray printing technique could be extended to an economical means of mass production for iEAP actuators.

## Figures and Tables

**Table 1:** Physical properties of the SWCNTs.

Outer Diameter	Purity	Length	Surface Area	Electric Conductivity	Ig/Id
<2 nm	>95 wt%	5–30 µm	> 490 m^2^/g	>100 S/cm	>20

## Data Availability

Data are contained within the article.
